# An unusual presentation of neuropathic pain following cervical spinal cord injury: a case report

**DOI:** 10.1186/s12883-020-01644-0

**Published:** 2020-02-18

**Authors:** Min Cheol Chang, Mathieu Boudier-Revéret, Yoo Jin Choo, Ming-Yen Hsiao

**Affiliations:** 1 Department of Physical Medicine and Rehabilitation, College of Medicine, Yeungnam University, Namku, Taegu, Republic of Korea; 2 Department of Physical Medicine and Rehabilitation, Centre hospitalier de l’Université de Montréal, 3840, Saint-Urbain St., Montreal, QC H2W 1T8 Canada; 3 Department of Physical Medicine and Rehabilitation, National Taiwan University Hospital, College of Medicine, National Taiwan University, Taipei, Taiwan

**Keywords:** Neuropathic pain, Spinal cord injury, Central cord syndrome, Occipital headache

## Abstract

**Background:**

We report a patient with unusual occipital neuropathic pain (at-level neuropathic pain) due to a small central cervical spinal cord injury (SCI).

**Case presentation:**

A 50-year-old man presented with severe bilateral occipital pain after falling from a height of 2 m, 2 weeks ago. The degree of pain was evaluated to be 9 out of 10 using the numeric rating scale (NRS). The nature of the pain was tingling, burning, and piercing, and hyperalgesia was present over the bilateral posterior head regions. Greater occipital nerve block with bupivacaine and dexamethasone was not effective. On axial T2-cervical magnetic resonance imaging (MRI), a focal high signal change was observed in the central portion of the spinal cord at the C2 level. We deliberated that the patient’s pain was due to the SCI observed on MRI, and after administration of oral medications, the NRS pain score reduced from 9 to 2.

**Conclusions:**

Neuropathic pain caused by SCI varies according to the location and degree of injury of the pain-related neural tracts; therefore, clinicians should closely observe the pain patterns and findings on imaging in patients with SCI to determine the cause of pain accurately.

## Background

Neuropathic pain is a difficult complication of spinal cord injury (SCI) to manage as it can be severely debilitating and can result in inactivity and psychological problems, such as depression and anxiety [1]. It arises as a direct consequence of lesions in the somatosensory system, especially the lateral spinothalamic tracts [2].

Neuropathic pain is suspected when the nature of the pain is shooting, electric, burning, itching, pricking, tingling, or cold, and the location of the pain is in a region of sensory disturbance [3]. Neuropathic pain due to SCI can be divided into two main types, at-level and below-level neuropathic pain [4]. At-level neuropathic pain is pain that occurs in a segmental or dermatomal pattern within the dermatome at the level of neurological injury and three dermatomes below this level. Below-level neuropathic pain refers to pain that presents diffusely caudal to the level of SCI, more than three dermatomes below the level of SCI.

In this study, we present a patient with unusual presentation of at-level neuropathic pain after SCI.

## Case presentation

**Fig. 1:**
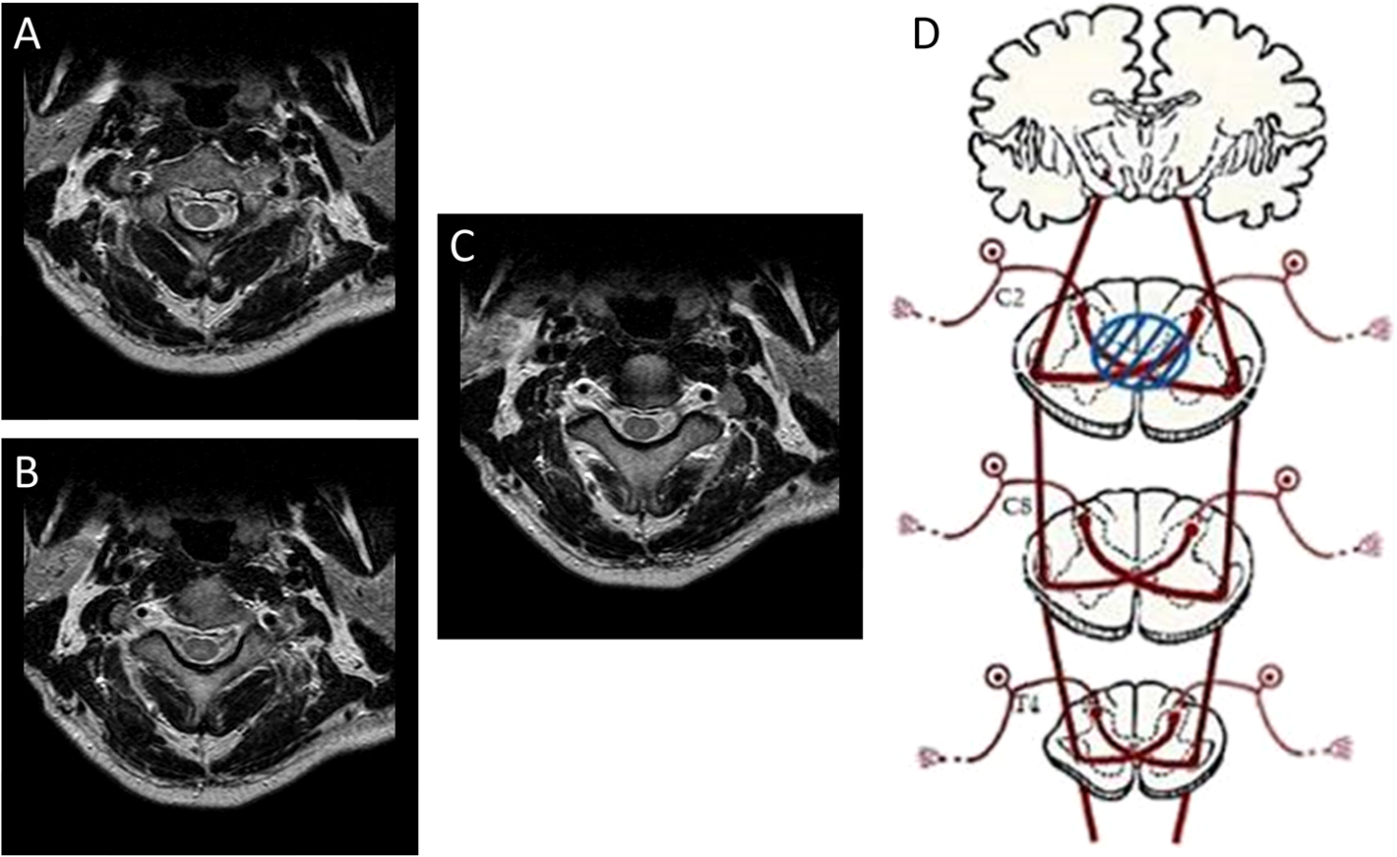
**a**, **b**, **c** Three sequential cranial to caudal axial T2-weighted cervical spine magnetic resonance images at the C2–3 disc-level reveal focal high signal intensity in the central portion of the spinal cord. **d** A schematic diagram of the lateral spinothalamic tracts and injured area in the cervical spinal cord. The area within the blue circle indicates the injured region

A 50 year-old-man visited the Department of Physical Medicine and Rehabilitation at a university hospital because of severe bilateral occipital pain after falling from a height of two meters, 2 weeks ago. The neck had flexed upon hitting the ground. His pain started immediately after the accident, and the numeric rating scale (NRS) score was 9 out of 10. The pain was tingling, burning, and piercing in nature with hyperalgesia over the bilateral posterior head regions. No sensory deficits were observed in the upper and lower limbs or trunk. Furthermore, there was no motor weakness. The deep tendon reflexes were normal in the upper and lower limbs. The patient’s anal tone and perianal sensation were intact. Ultrasound-guided greater occipital nerve block with 1.5 mL of 0.5% bupivacaine and 4 mg of dexamethasone was performed, and no short- or long-terms effects were not found. On axial T2-cervical magnetic resonance imaging (MRI) (1.5 T, Magetom Vision, Seimens, Erlangen, Germany; reconstrunction matrix = 216 × 152, field of view =140 × 140 mm^2^, echo time = 100 ms, repetition time = 3739 ms) performed 2 weeks post-injury, a focal high signal change was observed in the central portion of the spinal cord at the C2 level, with no bone fracture (Fig. 1). Also, mild central canal stenosis was presented at the C4–5 and C5–6 levels. The SCI manifested on cervical MRI seemed to have resulted in the patient’s pain. After administration of oral medications, including 150 mg of pregabalin twice daily, 75 mg/625 mg of tramadol/acetaminophen twice daily, and 10 mg of buprenorphine (skin patch) once weekly, the patient’s pain became bearable (NRS score: 2).

## Discussion and conclusions

The neurons of the lateral spinothalamic tract originate in the spinal dorsal root ganglia and enter the spinal cord via the posterior horn. Subsequently, they decussate across the anterior white commissure and ascend in the lateral spinothalamic tract on the contralateral side [5]. Damage of this neural tract from SCI can result in neuropathic pain at or below the level of the injury. In our patient, neuropathic pain following SCI occurred in the bilateral occipital regions, which corresponds to the C2 dermatome [6]. The neuropathic pain seemed to have resulted from the injury of the central portion of the spinal cord at the C2–3 level. Due to the lesion, only the bilateral lateral spinothalamic tracts receiving pain signals from the C2 dermatome were damaged in the decussation portion, without involving the lateral spinal neural tracts, such as the lateral spinothalamic tract leading to the brain after the decussation (containing afferent fibres below the lesion) and the corticospinal tract (Fig. 1). Consequently, following SCI from the fall, neuropathic pain at the level of the injury, which was at the C2 dermatome, developed in our patient, with no significant motor deficits. The condition of our patient corresponds to central cord syndrome. Usually in patients with central cord syndrome, motor weakness is manifested below the level of injury with sensory deficits. Because upper extremity motor fibres are located more centrally than lower extremity motor fibres, motor function of upper extremities is typically more severely impaired than lower extremities [7]. However, when the lesion size is small as in our patient, only bilateral pain and loss of tactile sensation at the affected level can be manifested without motor deficits.

Here, we reported a case of unusual occipital neuropathic pain due to a small central cervical SCI. As SCI can cause neuropathic pain in various aspects, depending on the location and degree of the injury of the pain-related neural tracts, clinicians should closely observe the pain patterns and findings on imaging in patients with SCI to determine the cause of pain accurately.

## Data Availability

Data sharing is not applicable to this article as no datasets were generated or analysed during the current study.

## Abbreviations

MRI: Magnetic resonance imaging
NRS: Numeric rating scale
SCI: Spinal cord injury

## References

[1] Burke D, Fullen BM, Stokes D, Lennon O (2017). Neuropathic pain prevalence following spinal cord injury: a systematic review and meta-analysis. Eur J Pain.

[2] Wasner G, Lee BB, Engel S, McLachlan E (2008). Residual spinothalamic tract pathways predict development of central pain after spinal cord injury. Brain.

[3] Colloca L, Ludman T, Bouhassira D, Baron R, Dickenson AH, Yarnitsky D, Freeman R, Truini A, Attal N, Finnerup NB, Eccleston C, Kalso E, Bennett DL, Dworkin RH, Raja SN (2017). Neuropathic pain. Nat Rev Dis Primers.

[4] Jang JY, Lee SH, Kim M, Ryu JS (2014). Characteristics of neuropathic pain in patients with spinal cord injury. Ann Rehabil Med.

[5] Nathan PW, Smith M, Deacon P (2001). The crossing of the spinothalamic tract. Brain.

[6] Lee DG, Chang MC (2017). Neck-to-shoulder pain as an unusual presentation of pulmonary embolism in a patient with cervical spinal cord injury: A case report. Medicine (Baltimore).

[7] Nowak DD, Lee JK, Gelb DE, Poelstra KA, Ludwig SC (2009). Central cord syndrome. J Am Acad Orthop Surg.

